# Communication Between a Dorsal and Ventral Ramus: A Conflict for Traditional Anatomical Teaching

**DOI:** 10.7759/cureus.2460

**Published:** 2018-04-10

**Authors:** Mayank Patel, Joe Iwanaga, Rod J Oskouian, R. Shane Tubbs

**Affiliations:** 1 Clinical Anatomy Research, Seattle Science Foundation; 2 Seattle Science Foundation; 3 Neurosurgery, Swedish Neuroscience Institute; 4 Neurosurgery, Seattle Science Foundation

**Keywords:** anatomy, cadaver, dissection, spinal nerve, cervical plexus, dorsal ramus, ventral ramus, lesser occipital nerve, greater occipital nerve

## Abstract

Counterintuitive to anatomic organization is the communication between a dorsal ramus and a ventral ramus, which are traditionally taught as serving specific anatomical fields. Herein, we discuss the cadaveric findings of such an example of neural intercommunication between the dorsal and ventral rami and review the potential embryology. While these two nerves are complementary in their function, it is not understood as to the reason for such an anatomical connection that breaches normal anatomical “laws.”

## Introduction

The lesser occipital nerve (LON) arises largely from the ventral ramus of the second cervical nerve and, sometimes, the ventral ramus of the third cervical nerve. It winds anterior to the accessory nerve and travels cranially along the posterior border of the sternocleidomastoid muscle. As it approaches the cranium, it pierces the deep fascia to enter the subcutaneous tissues of the scalp posterior to the auricle. The LON supplies the skin in the region and converges with other nerves in the region [1]. The function of the LON is cutaneous sensation behind the auricle and over the mastoid region [2]. The origin and course of the greater occipital nerve (GON) take a complex route to its terminal endings supplying the skin over the occiput. The GON arises from the dorsal ramus of the second cervical nerve and, possibly, the dorsal ramus of the third cervical nerve. The GON then courses between the obliquus capitis inferior and the semispinalis capitis muscles. It pierces the trapezius muscle or its fascia and ascends cranially with the occipital artery. The GON provides cutaneous innervation of the occiput as superiorly and anteriorly as the vertex of the skull [2]. The LON and GON serve two separate anatomical fields, i.e., a region served by a ventral ramus and a region served by a dorsal ramus. Traditionally, there is no overlap between the nerves serving these fields. However, the uncommon neural intercommunication between the LON and GON, as described below, offers an opportunity to discuss such overlap in the nervous system and posit the potential embryology involved.

## Case presentation

**Figure 1:**
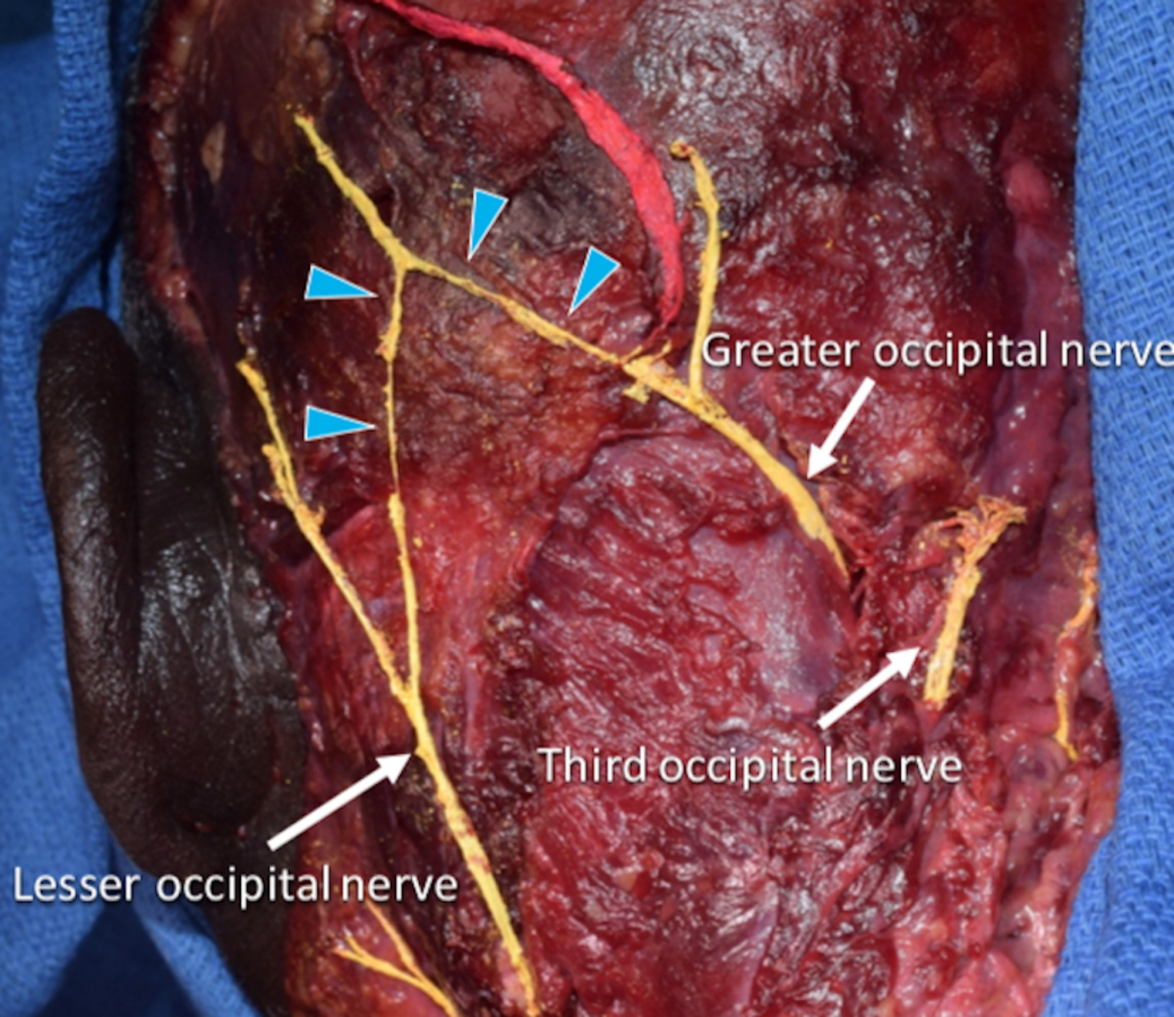
Cadaver presented herein illustrating the neural interconnection between the greater and lesser occipital nerves (arrowheads)

During the routine dissection of the left posterior-lateral neck in a fresh-frozen 74-year-old African-American male cadaver, an interconnection between the LON laterally and the GON medially was identified (Figure 1). The GON pierced the cranial tendon of the trapezius and gave rise to the medial and lateral branches. The medial branch ascended medial to the occipital artery, which pierced the tendon of the trapezius at the superior nuchal line and the lateral branch traveled latero-superiorly.The LON traveled along the posterior border of the sternocleidomastoid, ascended, and divided into the medial and lateral branches. The medial branch anastomosed with the lateral branch of the GON. No other anatomical anomalies were noted in the specimen relating to the posterior cervical or occipital regions. No gross findings of previous surgical intervention in the region dissected were identified.

## Discussion

The interconnection between the LON and GON is of interest, as this variation, to our knowledge, has not previously been discussed or presented as being unusual when illustrated. This neural variant has only been depicted in a few extant sources and these do not discuss the finding [3-5]. Our case clearly demonstrated an interconnection between the LON and GON. This adjoining of the nerve is derived from a dorsal ramus and one derived from a ventral ramus has relevant anatomical significance, as traditional anatomical teaching is that these are separate “systems” with the dorsal rami innervating only the deep “native” back muscles, the skin of the back, and facet joints. These two nerves, LON and GON, arise from opposite axes of the spinal cord but, interestingly, have forged an interconnection in the present case after reaching their respective position on the occiput. 

**Figure 2:**
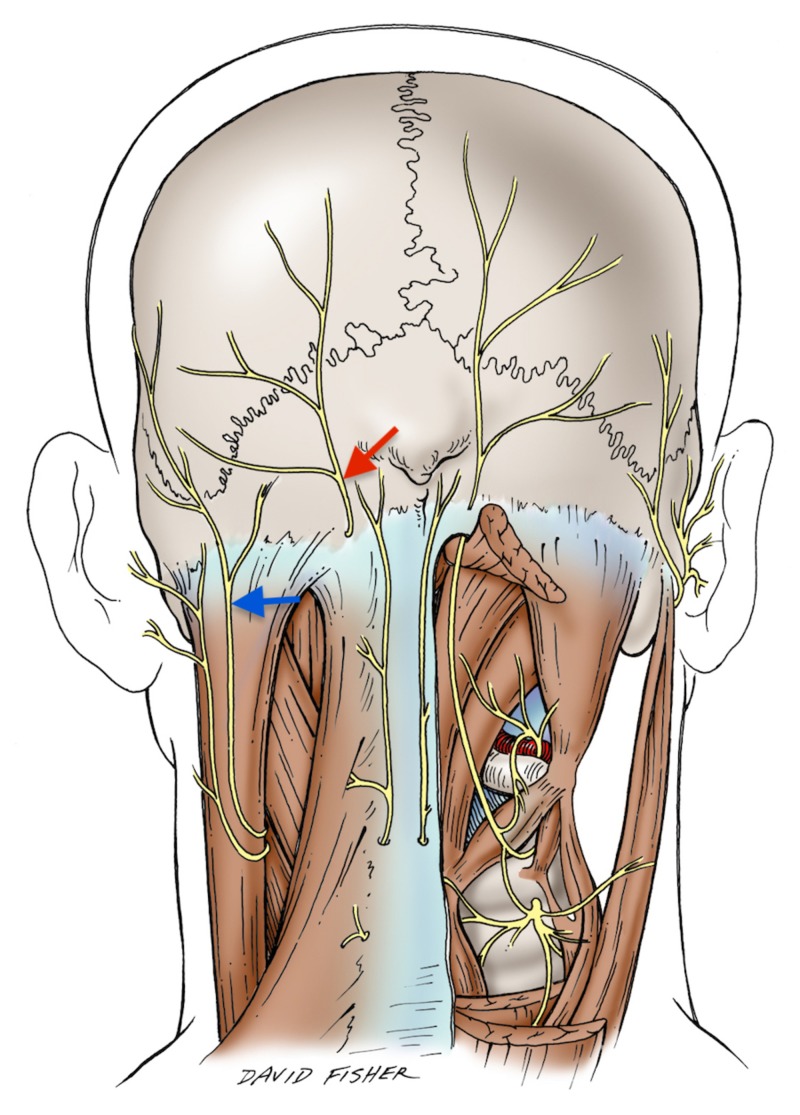
Schematic illustration of the greater and lesser occipital nerves Greater Occipital Nerve (Red Arrow) Lesser Occipital Nerve (Blue Arrow)

The existence of communication between the LON and the GON within axial lines is reported by another study mapping peripheral nerve communications [6]. However, the reason for such an interconnection between these two nerves is not clear and, to our knowledge, no hypotheses have been previously suggesting such an arrangement. In the schematic illustration (Figure 2), the proximity of the LON and GON are evident.

The development of the dorsal ramus and ventral ramus occurs in different time periods and from different structures during embryonic development. The idea that an interconnection between structures from different embryonic tissue has found a pathway to adjoin does not match conventional anatomical reasoning. Reviewing embryonic development on the 26^th^ day, the ventral motor somatic nerves begin to form from the ventral basal plate of the neural tube. These motor nerves begin to form prior to the formation of sensory nerves from the neural crest. Two days later, the neural crest begins forming sensory nerves to the dorsum, the lateroventral surface, and the viscera. There is also a development of the commissural neuron and association neuron, which interdigitate from the alar to the basal plate. Between five to seven weeks of development, there is an explosion of neurons developing from the basal plate, including preganglionic sympathetic motor neurons in the spinal nerve, postganglionic sympathetic motor neurons supplying smooth muscles, sweat glands, and viscera, and preganglionic sympathetic motor neurons supplying the next sympathetic trunk ganglion [7]. According to the embryonic timeline, the dorsal sensory nerves form after the ventral motor nerves, suggesting that there should not be any overlapping period during development where an interconnection could take place.

Dividing peripheral nervous system development into the somatic and splanchnic parts shows the importance of the dorsal and ventral rami during embryogenesis. The somatic development is divided into epimeres and hypomeres of the dermatomyotomes around the sixth week of embryonic development. The intrinsic back muscles will develop from the epimere portion, which is innervated by the dorsal rami. These intrinsic back muscles are also known as the epaxial muscles. The hypomere portion is innervated by the larger ventral rami and forms the hypaxial muscles, which include the anterior abdominal wall, upper limb, and lower limb muscles [7-8]. 

The dorsal and ventral rami are mixed nerves that supply different axial planes in humans. Therefore, an interconnection at the development of the epaxial and hypaxial muscles and its overlying dermis could occur during the sixth week of embryogenesis. An extensive review of the interconnections between the dorsal and ventral rami could rearrange traditional anatomic teachings in relation to the arrangement of the nervous system and separate innervation fields.

## Conclusions

Such a cadaveric finding leaves many questions unanswered about the function of this type of an interconnection. The interconnection found here is one example of ventral and dorsal rami uniting to subserve a common anatomical area and is at odds with classical anatomical teaching.
